# Epidemiology of mental health problems among patients with cancer during COVID-19 pandemic

**DOI:** 10.1038/s41398-020-00950-y

**Published:** 2020-07-31

**Authors:** Yuanyuan Wang, Zhizhou Duan, Zikun Ma, Yize Mao, Xiyuan Li, Amanda Wilson, Huiying Qin, Jianjun Ou, Ke Peng, Fangjian Zhou, Chaofeng Li, Zhuowei Liu, Runsen Chen

**Affiliations:** 1 Department of Psychiatry & Mental Health Institute of the Second Xiangya Hospital, Central South University, Chinese National Clinical Research Centre on Mental Disorders (Xiangya), Chinese National Technology Institute on Mental Disorders, Hunan Key Laboratory of Psychiatry and Mental Health, Changsha, Hunan China; 2 Division of Psychology, Faculty of Health and Life Sciences, De Montfort University, Leicester, UK; 3 School of Health Sciences, Wuhan University, Wuhan, China; 4 Sun Yat-sen University Cancer Center, State Key Laboratory of Oncology in South China Collaborative Innovation Center for Cancer Medicine, 510060 Guangzhou, China; 5 Department of Urology, Sun Yat-sen University Cancer Center, Guangzhou, China

**Keywords:** Diseases, Psychiatric disorders

## Abstract

The current study aimed to explore mental health problems in patients diagnosed with cancer during the COVID-19 pandemic. A cluster sampling, cross-sectional survey with 6213 cancer patients was conducted in one of the largest cancer centers in China. The socio-demographic and clinical characteristics, psychosomatic conditions, interpersonal relationships and social support, COVID-19 infection-related psychological stress, and mental health status were measured. Medical conditions were extracted from patients’ electronic healthcare records. Among the 6213 cancer patients, 23.4% had depression, 17.7% had anxiety, 9.3% had PTSD, and 13.5% had hostility. Hierarchical liner regression models showed that having a history of mental disorder, excessive alcohol consumption, having a higher frequency of worrying about cancer management due to COVID-19, having a higher frequency feeling of overwhelming psychological pressure from COVID-19, and having a higher level of fatigue and pain were the predominant risk factors for mental health problems in cancer patients. However, there were only 1.6% of them were seeking psychological counseling during COVID-19. We also revealed the protective factors associated with lower risk of mental health problems among cancer patients. The present study revealed a high prevalence of mental health problems and gaps in mental health services for cancer patients, which also indicated high distress from COVID-19-elevated risks. We call for systematic screening of mental health status for all cancer patients, and developing specific psychological interventions for this vulnerable population.

## Introduction

Cancer diagnosis and treatment can give rise to considerable mental health issues for individuals, such as anxiety and depression^1^. Compared with healthy populations, cancer patients are at a higher risk of mental health problems^2–4^. According to a previous systematic estimation of cancer prevalence in China, cancer incidences are around 3.37 million and the mortality rate is 2.11 million people per year^5^. Previous research in China also shows that 15.8% of cancer patients had clinical symptoms of a mental disorders, with depression, anxiety, psychotic symptoms, and stress-related disorders accounting for 13.3%, 10.2%, 2.8% and 1.4%, respectively^6^. It has been estimated that approximately one-third of cancer patients are affected by mental disorders, with depression being most prominent^7–9^. Left untreated mental health problems in cancer patients can led to destructive consequences including decreased treatment adherence, decreased survival rate, increased healthcare cost, and poor quality of life^10–13^. Research has shown that cancer patients with severe mental health problems are more likely to be hospitalized and die in 12 months after their cancer diagnosis when compared to patients without or with less severe mental health problems^14^.

Currently, the COVID-19 pandemic brings mental health distress on a global level^15–17^. In a recently published study of the general population in China during the COVID-19 outbreak, people reported suffering from mental health problems with the highest percentages accounted for by stress, anxiety, and depression, which were 8.1%, 28.8%, and 16.5%, respectively^17^. Considering the vulnerability of the population, it remains uncertain the impact and consequences of the COVID-19 infection on individuals with underlying health conditions^18^. Another recent published study in China has indicated that psychiatric patients suffered from more severe psychiatric symptoms during the COVID-19 with higher scores for a range of symptoms including, anxiety, depression, stress, post-traumatic stress disorder, insomnia, poor physical health, anger, impulsivity, and higher suicidal ideations^19^. Moreover, the COVID-19 pandemic has caused inconveniences for individuals who need regular assessments at hospitals, such as the lack of treatment due to the shortage of healthcare resources. Therefore, the COVID-19 pandemic is likely to create or exaggerate mental health problems in cancer patients.

The current study aimed to provide a comprehensive mental health status screening in a large cluster sample of patients with various types of cancer during the COVID-19 pandemic. We assessed the impact of socio-demographic and clinical characteristics, distress, and inconveniences caused by the COVID-19 pandemic, as well as social support and somatic conditions on mental health outcomes including depression, anxiety, hostility, and post-traumatic stress disorder (PTSD) symptoms. We detected the prevalence, risk factors, and protective factors contributing to the different mental problems separately. The identification of risk factors on mental health problems is constitutive for the early detection of at-risk groups of cancer patients. It is also important for management of psychosocial interventions to improve patients’ long-term mental health condition.

## Methods

### Participants

This was a cross-sectional study that recruited cancer patients by cluster sampling from 9 April to 19 April, 2020 in Sun Yat-sen University Cancer Center, China. Sun Yat-sen University Cancer Center is one of the largest and leading radiotherapy centers in the world. We invited 9978 cancer patients who attended the cancer center by mobile message with a website hyperlink. Of those invited 6537 patients completed this survey, reaching a response rate of 65.5%. There were 324 patients excluded from the data analysis due to their responses missing key information and/or providing invalid information. Those who participated were given an online information sheet and consent form to sign, they were assured anonymity by assigning them a participant number. All the patients had a diagnosis of a malignant tumor according to International Statistical Classification of Diseases (10th Revision [ICD-10] codes C00-C97)^20^ and World Health Organization (WHO) classification of tumors^21^. All participants were asked to provide consent to access their medical record number in order to confirm their medical status. The ethics committee of Sun Yat-sen University Cancer Center approved this study (ref no. B2020-081-01).

### Measures

#### Socio-demographic and clinical characteristics

Socio-demographic and clinical characteristics included sex, age, residence location, annual family income, education, marital status, employment status, and history of mental disorder (diagnosed by psychiatrist). Excessive alcohol intake was measured by asking a single item “Have you experienced the following symptoms due to drinking alcohol, including dizziness, headache or drowsiness, etc. during the COVID-19 pandemic” based on a 4-point Likert scale from 1 never to 5 very often (three to five times per month). Medical conditions (such as time since cancer diagnosis) of cancer patients were extracted from the patients’ electronic healthcare records.

#### Factors related to COVID-19

Participants were asked about the frequency of worrying about cancer management/control due to COVID-19, and frequency of receiving information and news related to COVID-19 based on a 5-point Likert scale from 1 never to 5 very often. Participants were asked the barriers to accessing treatment during COVID-19, which they could choose from 1 no barriers to 4 severe barriers. We also asked participants about frequency of feeling an overwhelming psychological pressure due to the COVID-19 pandemic, with answers ranging from 1 never to 5 very often.

#### Psychosomatic characteristics

Participants were asked about the level of physical fatigue and pain intensity via a Visual Analogue Scale (VAS), they could choose an appropriate number ranging from 0 (no) to 10 (extreme). This scale has been widely used in cancer-related fatigue and pain research^22–24^. In addition, participants were asked about their quality of life and the degree of satisfaction about their physical health using the World Health Organization Quality of Life-Brief (WHOQOL-BRIEF) quality of life assessment. Participants could choose an answer that ranged from 1 (extremely unsatisfied) to 5 (extremely satisfied)^25^. Sleep quality was assessed by self-report items based on the DSMIV-Insomnia Criteria, where participants are asked three questions on identify the difficulties of initiating sleep, maintaining sleep, and early morning awakening. If participants reported one of these items they were defined as having poor quality of sleep. This definition of insomnia has been widely use in previous psychiatric studies^26,27^.

#### Social support and interpersonal relationships

Participants were asked about the quality of relationships with friends and family members, answer ranged from 1 (very bad) to 5 (very good). As for social support, participants were asked whether they were getting enough social support from the community during the COVID-19 pandemic, answers ranged from 1(no) to 4 (very much).

#### Mental health outcomes

Anxiety was measured by the Generalized Anxiety Disorder-7 (GAD-7), which consists of 7 items on a 4-point Likert scale, with higher total score indicating severe anxiety symptoms^28^. In the current study, a cut-off score of equal to and above 7 indicated high risk of clinical anxiety^29^. Depression was measured by the Patient Health Questionnaire-9 (PHQ-9), using a 9-item 4-point Likert scale, with higher total scores indicating a higher level of depression symptoms; a cut-off score of 7 indicated high risk of clinical depression^30,31^. Hostility was measured by hostility subscale of the Brief Symptom Inventory (BSI), which consists of five items with rating from 0 (not at all) to 4 (extremely);a cut-off scores of 4 indicated higher risk of hostility^32,33^. COVID-19-related PTSD was measured by the Impact of Events Scale-Revised (IES-R), which is a 22-item 5-point Likert scale; higher total scores indicate severe PTSD symptoms, with a cut-off score of 33 to recommended PTSD diagnosis^34^. IES-R has been used in previous COVID-19 research in China^35,36^. In addition, we also assessed the prevalence of psychological or psychiatric counseling service use and attitudes.

### Statistical analysis

The sample characteristics were presented using mean and standardized deviation (SD) for continuous variables and using percentage for categorical variables. Hierarchical liner regression models were applied to explore the contribution of socio-demographic characteristics, factors related to COVID-19, somatic characteristics, and support on mental health outcomes, and the increasing of *R*^2^ indicted the explanation of the independent variables on the dependent variables, which further verified the importance of the independent variables on dependent variables. In addition, using robust test to confirm the hierarchical liner regression models. All analyses were conducted using SPSS package, Version 20.0, and R software, version 3.6.1, Stata software, version 12.0 and *p* < 0.05 (two-tails) were considered to have statistical significance.

## Results

### Socio-demographic and clinical characteristics of the sample

There were 6213 cancer patients who completed the survey. As in Table 1, around half of the patients were male (52.8%), and the average age of the patients were 50.57 (SD = 13.28) years old. The majority of the patients (87.8%) were married and 2.3% had history of mental disorder. The majority of the patients (68.9%) lived in urban areas, less than half (39.2%) had college or higher education, and 19.4% had family income equal or above 150, 000 Chinese Yuan (~21,000 US dollars). Over one-third of the patients (36.6%) had high barriers to continue cancer treatment due to inconveniences (e.g., transport) caused by COVID-19.

**Table 1:** Demographic and clinical characteristics of cancer patients (*N* = 6213).

Variables	Number (*n*)	Percent (%)
Age (mean ± SD)	50.57 ± 13.28	
Sex		
Men	3278	52.8
Women	2935	47.2
Educational level		
Junior school or lower	2033	32.7
High school	1744	28.1
College or above	2436	39.2
Family annual income		
<60,000	3343	53.8
60,000–150,000	1667	26.8
>150,000	1203	19.4
Residence place		
Rural	1933	31.1
Urban	4280	68.9
Marital status		
Married	5452	87.8
Unmarried/others	761	12.2
Employment status		
Full-time job	2291	36.9
Part-time job	262	4.2
Retire	1631	26.3
Unemployed	2029	32.7
History of mental disorder		
Depression	88	1.4
Bipolar disorder	9	0.1
The generalized anxiety disorder	66	1.1
Phobia	15	0.2
Eating disorder	40	0.6
Obsessive-compulsive disorder	27	0.4
PTSD	10	0.2
Schizophrenia	7	0.1
Panic disorder	14	0.2
Substance dependence	12	0.2
Others	15	0.2
Had barriers to continuing treatment		
No	2277	36.6
Yes	3936	63.4
Time since diagnosis		
1–4 weeks ago	518	8.3
1–3months ago	755	12.2
3–6monthes ago	1076	17.3
6–12 months ago	1107	17.8
1–3years ago	1626	26.2
3–5 years ago	585	9.4
More than 5 years	546	8.8
Received treatment		
Surgery (yes)	2431	39.1
Radiotherapy (yes)	1871	30.1
Chemotherapy (yes)	3758	60.5
Other treatments (yes)	506	8.1

*CNY* Chinese Yuan, *PTSD* post-traumatic stress disorder.

### Mental health outcomes

Among the whole sample 1101 (17.7%) who had anxiety symptoms, 1456 (23.4%) had depression symptoms, 578 (9.3%) had PTSD symptoms and 839 (13.5%) had hostility symptoms. The results revealed that there were shared risk factors across different mental health problems. As in Table 2, the following risk factors included: having a history of mental disorder, excessive drinking, higher frequency of worrying about disease management due to COVID-19, increasing psychological pressure from COVID-19, having higher level of fatigue intensity, and having higher level of pain intensity. These risk factors were the predominant risk factors across anxiety, depression, hostility, and PTSD. Beside the shared common risk factors, the results identified unique contributing risk factors for individual mental problem. Inconveniences to go out for follow-up treatment (*b* = 0.043, *p* < 0.01) was associated with higher risk of depression; and longer time since diagnosis (*b* = 0.035, *p* < 0.01) and higher frequency of receiving COVID-19 information and news (*b* = 0.021, *p* < 0.05) were associated with a higher level of PTSD symptoms.

**Table 2:** The associations between different mental health outcomes and risky factors (*N* = 6213).

Variables	Model of anxiety				Model of depression				Model of hostility				Model of PTSD			
	Model 1	Model 2	Model 3	Model 4	Model 1	Model 2	Model 3	Model 4	Model 1	Model 2	Model 3	Model 4	Model 1	Model 2	Model 3	Model 4
*Socio-demographics and clinical characteristics*																
Age	−0.020	−0.019	−0.028**	−0.025**	−0.002	−0.001	−0.014	−0.011	−0.067***	−0.066***	−0.074***	−0.068***	0.019	0.021	0.009	0.013
Sex (men)	−0.060***	−0.031**	−0.031**	−0.032**	−0.031**	−0.007	−0.006	−0.006	−0.027*	−0.007	−0.006	−0.007	−0.095***	−0.064***	−0.057***	−0.058***
Residence place (urban)	0.014	0.018	0.002	0.001	0.032*	0.035**	0.016	0.015	0.021	0.022	0.008	0.006	0.009	0.009	−0.003	−0.006
Annul family income (≥150,000)	−0.034**	−0.018	−0.008	−0.006	−0.041**	−0.024*	−0.013	−0.012	−0.010	0.001	0.007	0.010	−0.029*	−0.017	−0.007	−0.006
Marital status (married)	−0.017	−0.019	0.001	0.001	−0.034**	−0.034**	−0.013	−0.012	−0.001	−0.001	0.017	0.018	0.009	0.005	0.016	0.016
Educational level (college or higher)	−0.002	−0.008	−0.013	−0.015	−0.003	−0.010	−0.016	−0.018	0.018	0.011	0.006	−0.002	0.016	0.005	0.001	−0.003
Employment status (yes)	−0.087***	−0.060***	−0.032**	−0.030**	−0.109***	−0.083***	−0.049***	−0.048***	−0.066***	−0.047***	−0.024	−0.021	−0.036*	−0.013	0.007	0.011
History of mental disorder (yes)	0.145***	0.111***	0.053***	0.050***	0.143***	0.111***	0.043***	0.040***	0.135***	0.111***	0.059***	0.051***	0.153***	0.119***	0.069***	0.065***
Excessive alcohol intake (yes)	0.083***	0.067***	0.041***	0.038***	0.068***	0.052***	0.021*	0.019*	0.117***	0.105***	0.081***	0.074***	0.107***	0.087***	0.063***	0.060***
Time since diagnosis	−0.054***	−0.023	−0.015	−0.023*	−0.059***	−0.030*	−0.019***	−0.024**	0.006	0.027**	0.033**	0.018	0.014*	0.040***	0.046***	0.035***
Received treatment (yes)	−0.072***	−0.052***	−0.047***	−0.048***	−0.037**	−0.015	−0.008	−0.008	0.015	0.001	−0.001	0.001	−0.015	−0.001	−0.002	−0.002
*COVID-19-related risks*																
Frequency of worrying about disease management due to COVID-19		0.245***	0.104***	0.103***		0.235***	0.064***	0.063***		0.160***	0.035*	0.033*		0.169***	0.054***	0.052***
Frequency of receiving COVID-19 information (any sources)		−0.038**	−0.029**	−0.027**		−0.022	−0.011	−0.010		−0.005	0.001	0.004		0.012	0.020*	0.021*
Barriers to manage cancer caused by COVID-19		0.039*	0.003	0.004		0.049**	0.006	0.006		0.049*	0.016	0.018		0.052**	0.024	0.026
Barriers to continue cancer treatment caused by COVID-19		0.050**	0.017	0.015		0.085***	0.045***	0.043**		0.030	0.003	−0.002		0.041*	0.019	0.016
Increasing psychological pressure caused by COVID-19		0.263***	0.160***	0.150***		0.223***	0.102***	0.095***		0.189***	0.094***	0.076***		0.319***	0.234***	0.221***
*Psychosomatics factors*																
The level of fatigue			0.262***	0.251***			0.300***	0.292***			0.288***	0.265***			0.198***	0.184***
The level of pain			0.066***	0.069***			0.111***	0.113***			0.047**	0.051***			0.055***	0.059***
Satisfied with physical health			−0.052***	−0.049***			−0.072***	−0.071***			−0.010	−0.008			0.004	0.008
Quality of life			−0.155***	−0.133***			−0.155***	−0.140***			−0.121***	−0.075***			−0.076***	−0.047***
Sleep quality			−0.164***	−0.158***			−0.207***	−0.203***			−0.152***	−0.140***			−0.259***	−0.251***
*Support*																
Social support				−0.003				0.002				0.006				0.002
Relationships with friends				−0.025*				−0.007				−0.005				−0.062***
Relationships with family members				−0.068***				−0.062***				−0.203***				−0.068***
*AdjustR*^2^	0.054***	0.277***	0.479***	0.485***	0.049***	0.261***	0.553***	0.556***	0.044***	0.159***	0.331***	0.364***	0.046***	0.270***	0.428***	0.437***

*PTSD* post-traumatic stress disorder*.*

*p < 0.05.

**p < 0.01.

***p < 0.001.

Besides risk factors, results also revealed the protective factors associated with lower risk of mental health problems, including better quality of life and good relationships with family member. Younger age (*b* = −0.028, *p* < 0.01), male sex (*b* = −0.031, *p* < 0.01), being employed (*b* = −0.030, *p* < 0.01), longer time since diagnosis (*b* = −0.023, *p* < 0.05), receiving treatment (*b* = −0.048, *p* < 0.001), higher frequency of receiving COVID-19 information and news (*b* = −0.027, *p* < 0.01), satisfaction with personal health (*b* = −0.049, *p* < 0.001), good sleep quality (*b* = −0.158, *p* < 0.001), and having good relationships with friends (*b* = −0.025, *p* < 0.05) were associated with lower risk of anxiety. Having been employed (*b* = −0.048, *p* < 0.001), longer time since diagnosis (*b* = −0.024, *p* < 0.01), and good sleep quality (*b* = −0.203, *p* < 0.001) were associated with lower levels of depression. Younger age (*b* = −0.068, *p* < 0.001) was a protective factor against hostility. Male sex (*b* = −0.058, *p* < 0.001), good sleep quality (*b* = −0.251, *p* < 0.001), and good relationships with friends (*b* = −0.062, *p* < 0.001) were associated with lower levels of PTSD symptoms. The four model of adjust *R*^2^ also showed the effect of factors related to COVID-19 towards anxiety (0.054–0.277), depression (0.049–0.261), hostility (0.044–0.159), and PTSD symptoms (0.046–0.270).

**Fig. 1:**
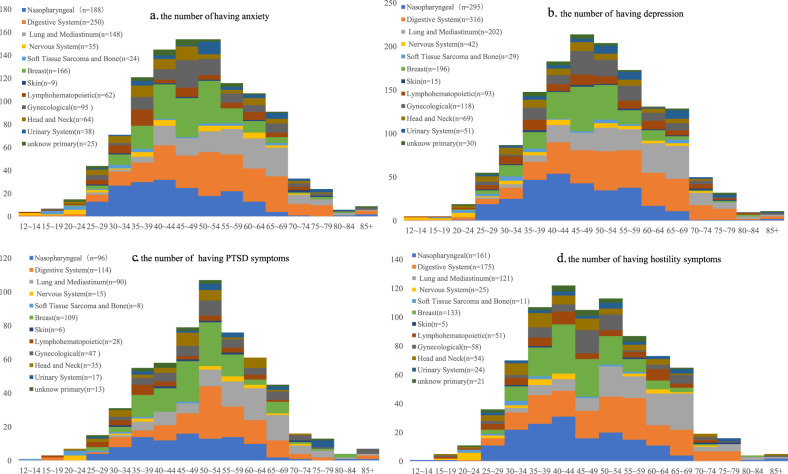
The prevalence of mental health problems among different type of cancers. The number of having mental disorders (depression, anxiety, PTSD and hostility) in different age group among different type of cancers.

Considering the higher prevalence of mental health problems in cancer patients, only 1.6% of them were seeking help for psychological counseling. Around half of the total patients, 2989 (48.1%), did not pay attention to online mental health services and only 696 (11.2%) considered online mental health services as helpful. As in Fig. 1, patients with digestive system cancer and breast cancer showed a higher proportion of having mental health problems. In detail, the patients with digestive system cancer accounted for 22.7% anxiety, 21.7% depression, 19.7% PTSD, and 20.9% hostility. The proportion of the breast cancer patients with the same mental health problems were 15.1%, 13.5%,18.9%, 15.9%, respectively. Being 45–54 years old as a patient also contributed to a relatively larger proportion of mental health problems.

In addition, compared with males (Table 3), the cancer patients who identified as female had a higher frequency of worrying about disease management due to COVID-19 (*t* = 2.13, *p* < 0.05), increasing psychological pressure caused by COVID-19 (*t* = 6.65, *p* < 0.001), and lower sleep quality (*t* = −4.15, *p* < 0.001).

**Table 3:** The differences of mental health outcomes, separated by male and female (*N* = 6213).

Variables	Total		Male		Female		*T*	*P*
	Mean	SD	Mean	SD	Mean	SD		
*COVID-19-related risks*								
Frequency of worrying about disease management due to COVID-19	2.36	1.13	2.34	1.13	2.40	1.13	2.13	**0.034**
Frequency of receiving COVID-19 information (any sources)	3.66	1.10	3.65	1.12	3.67	1.09	0.85	0.39
Barriers to manage cancer caused by COVID-19	1.97	0.76	1.98	0.76	1.96	0.76	−1.11	0.27
Barriers to continue cancer treatment caused by COVID-19	1.92	0.77	1.93	0.77	1.91	0.77	−1.33	0.19
Increasing psychological pressure caused by COVID-19	2.44	0.94	2.37	0.95	2.53	0.93	6.65	**<0.001**
*Psychosomatics factors*								
The level of fatigue	4.28	2.51	4.24	2.47	4.33	2.56	1.47	0.14
The level of pain	3.03	2.38	3.02	2.37	3.05	2.39	0.58	0.56
Satisfied with physical health	2.75	0.88	2.75	0.89	2.75	0.88	−0.17	0.87
Quality of life	3.30	0.74	3.30	0.74	3.30	0.75	0.04	0.97
Sleep quality	4.71	1.28	4.78	1.26	4.64	1.28	−4.15	**<0.001**
*Support*								
Social support	1.89	0.79	1.88	0.78	1.89	0.81	0.54	0.59
Relationships with friends	1.83	0.83	1.82	0.83	1.83	0.83	0.40	0.69
Relationships with family members	1.56	0.75	1.55	0.74	1.58	0.75	1.43	0.15

Bold values identify statistical significancce (*p* < 0.05)

## Discussion

This is the first large-scale screening for mental health problems and associated factors among patients with various types of cancer during COVID-19 pandemic. The findings suggest a need for screening for the prominence of mental health conditions after cancer diagnosis. The prevalence of depression, anxiety, PTSD and hostility were 23.4%, 17.7%, 9.3%, and 13.5% respectively. As stated in the introduction, during the COVID-19 crisis, two studies conducted by Wang and colleagues in the Chinese general population showed that 16.5% had depressive symptoms, 28.8% had anxiety symptoms, and 8.1% had stress symptoms, which was consistent over time^16,17^. Another study conducted by Hao and colleagues showed that in a healthy Chinese population the prevalence of anxiety symptoms were 2.7%, depressive symptoms were 0.9%, and stress symptoms were 0.9%^19^. The current results indicate that during the COVID-19 crisis cancer patients tend to overall have higher level of mental health symptoms compared to general Chinese populations. However, anxiety symptoms were higher in the Wang et al. study and lower in the Hao et al. study. Although the prevalence of mental problems varied among different settings, regions, different diagnostic tools, and different cut-off score for scales, when compared with previous research on cancer patients in China, our study showed a higher rate of poor mental health outcomes for the outbreak of COVID-19^6,37^. For example, Song and Li^6^ found that the prevalence of depression, anxiety and PTSD was 13%, 10.2%, and 1.4%, respectively, among 2279 cancer patients from nine medical centers in China. Moreover, it further indicated that the current COVID-19 pandemic added an extra burden for cancer patients, increasing their mental health problems.

As stated, the current results revealed a high prevalence of mental health problems in cancer patients, with an extremely small proportion of patients seeking help for psychological counseling. Researchers have noted the gap between the high prevalence of mental disorders and low prevalence of mental health treatment, and have highlighted that many cancer patients with mental health problems do not receive adequate treatment accordingly^12,38^. During the COVID-19 pandemic, it is recommended high risk groups be identified for psychological morbidities and screening be improved to provide quick, cost effective psychological interventions via online platforms to manage symptoms^39^. Policy makers should plan psycho-oncological care for cancer patients, especially using a combine effort from all staffing levels who deliver cancer care to deliver screening, including oncologists, psychiatrists, nurses etc^7^. Moreover, it is recommended by American Society of Clinical Oncology that all patients diagnosed with cancer should be screened for symptoms of depression and anxiety^40^. Alongside the recommendation from American Society of Clinical Oncology^40^, we call for all patients being provided with mental health assessment after cancer diagnosis. Considering the lack of time and resources for personalized screenings, researchers have recommended programs such as Mental Health and Dynamic Referral for Oncology (MHADRO) to assist with screening for mental health problems in cancer patients^41^. MHADRO is a computerized assessment that could provide a personalized summary report of a patient’s psychological functions, and could assist the mental health treatment initiation^42^. We strongly recommended oncology clinics to provide necessary and timely mental health screening for cancer patients using all available and accessible methods. In particular, any online methods available in light of social distancing measures.

The current findings have significant clinical implications for detecting and treating different mental health problems in cancer patients. Remarkably, our results revealed both shared and unique risk factors and protective factors for different mental health problems. The identification of mental health risk factors could facilitate efficient screening and management of mental health problems in cancer patients^41^. We found having a history of mental disorder, excessive drinking, and having higher levels of fatigue and pain were the predominant risk factors for mental health problems in cancer patients. Consistent with previous research^14^, we found that more severe mental health problems in cancer patients were associated with somatic comorbidities, especially in terms of pain and fatigue. During the COVID-19 pandemic, there were more than one-third of patients who reported having some barriers to continue their cancer treatment due to inconveniences caused by COVID-19. Our results showed that cancer patients who had a higher frequency of worry about disease management due to COVID-19 and increased psychological pressure due to COVID-19 were at a higher risk of mental health problem. As emphasized by Calvo et al.^43^, health surveillance and monitoring is an important part of maintaining wellbeing during the COVID-19 pandemic. It is necessary to provide health services to cancer patients during COVID-19, in order to prevent the predictable decline of cancer conditions, possible hospitalization, as well as prevent health system overload and health control crisis.

We also identified predominant protective factors that lowered the risk of mental health problems, including having a satisfied quality of life and good relationships with family members. Low level of support was a significant predictor for distress in cancer patients^41^. Our results indicate that good family relationships had a positive influence for patients’ mental health. During COVID-19, family members could enhance their relationship with the patients to help them achieve a better mental health status. How to do this during a time of social distancing is important, with considerations for online conversations and playing joint games as possible alternatives. In the results, we found that a higher frequency of receiving COVID-19 information and news was associated with a lower risk of anxiety. This could due to Chinese news and media emphasizing the information about pandemic control, offering strategies for self-protection, and providing contact information for people who were in need.

The current study has several limitations. First, the causality in variables cannot be established due to the nature of cross-sectional design. Future longitudinal studies are needed for an in-depth understanding of the consequences of mental health problems in cancer patients. A longitudinal study of the general population^17^ showed that there was no significant change in anxiety, depression, and stress between the outbreak of COVID-19 and 4 weeks later. We would expect similar results in the cancer patients. Second, mental health problems were assessed using self-report questionnaires. We recommended the use of clinical interviews performed by psychiatrists in follow-up studies to provide more detail on the important factors of mental health problems. Moreover, the care restrictions caused by COVID-19 is based on self-reported answer, which could only reflect patients’ subjective perspectives. Third, the current study did not cover all the variables related to mental health problems. For example, a previous study showed that a lower income was associated with depression in cancer patients^44^. This study was unable to measure any changes in household income due to COVID-19, this is a point for further research. Finally, cancer patients with severe mental health disorders may stop attending hospitals for cancer treatment, therefore, it is possible that we underestimated the prevalence of mental health problems in patients.

## Conclusion

In conclusion, the current study raised vital concerns of the high prevalence of mental health problems in cancer patients during COVID-19. It is crucial to implement systematic mental health screening for all patients after cancer diagnosis. During a pandemic outbreak, mental health care attention and resources should be placed as a priority for cancer patients in order to help them to cope and prevent the decline of their mental health status.
